# Epithelial Galectin-3 Induced the Mitochondrial Complex Inhibition and Cell Cycle Arrest of CD8^+^ T Cells in Severe/Critical COVID-19

**DOI:** 10.3390/ijms241612780

**Published:** 2023-08-14

**Authors:** Yudie Wang, Cheng Yang, Zhongyi Wang, Yi Wang, Qing Yan, Ying Feng, Yanping Liu, Juan Huang, Jingjiao Zhou

**Affiliations:** 1 Department of Biology and Genetics, College of Life Sciences and Health, Wuhan University of Science and Technology, Wuhan 430065, China; 2 Department of Hematology, Maternal and Child Health Hospital of Hubei Province, Tongji Medical College, Huazhong University of Science and Technology, Wuhan 430070, China

**Keywords:** COVID-19, CD8^+^ T cell, cell cycle arrest, mitochondrial complex genes, galectin-3

## Abstract

Previous research suggested that the dramatical decrease in CD8^+^ T cells is a contributing factor in the poor prognosis and disease progression of COVID-19 patients. However, the underlying mechanisms are not fully understood. In this study, we conducted Single-cell RNA sequencing (scRNA-seq) and single-cell T cell receptor sequencing (scTCR-seq) analysis, which revealed a proliferative-exhausted *MCM*^+^*FASLG*^low^ CD8^+^ T cell phenotype in severe/critical COVID-19 patients. These CD8^+^ T cells were characterized by G2/M cell cycle arrest, downregulation of respiratory chain complex genes, and inhibition of mitochondrial biogenesis. CellChat analysis of infected lung epithelial cells and CD8^+^ T cells found that the galectin signaling pathway played a crucial role in CD8^+^ T cell reduction and dysfunction. To further elucidate the mechanisms, we established SARS-CoV-2 ORF3a-transfected A549 cells, and co-cultured them with CD8^+^ T cells for ex vivo experiments. Our results showed that epithelial galectin-3 inhibited the transcription of the mitochondrial respiratory chain complex III/IV genes of CD8^+^ T cells by suppressing the nuclear translocation of nuclear respiratory factor 1 (NRF1). Further findings showed that the suppression of NRF1 translocation was associated with ERK-related and Akt-related signaling pathways. Importantly, the galectin-3 inhibitor, TD-139, promoted nuclear translocation of NRF1, thus enhancing the expression of the mitochondrial respiratory chain complex III/IV genes and the mitochondrial biogenesis of CD8^+^ T cells. Our study provided new insights into the immunopathogenesis of COVID-19 and identified potential therapeutic targets for the prevention and treatment of severe/critical COVID-19 patients.

## 1. Introduction

Since the emergence of severe acute respiratory syndrome coronavirus 2 (SARS-CoV-2) in late 2019, it has triggered a pandemic of acute respiratory disease known as “coronavirus disease 2019” (COVID-19). Emerging variants of SARS-CoV-2 are still ravaging many countries, causing breakthrough infections and repeated infections, which are costly to public health and global economics [1]. COVID-19 patients include mild, moderate, and even severe or critical cases, each with distinct metabolic and immune profiles [2,3]. The dysregulated host immune responses to SARS-CoV-2 infection contributed to the persistence of the virus, which play important roles in the strong inflammatory response and consequent lung injury and multi-organ damage, and put patients at high risk for severe/critical COVID-19 [4].

Effective control of the acute viral infection requires the robust expansion of effector CD8^+^ T cells. However, clinical studies of circulating immune cells and lung samples have documented a sharp decrease in CD8^+^ T cells in severe/critical COVID-19 patients [5,6]. Recent studies have shown that CD8^+^ T cells from severe/critical COVID-19 patients exhibit impaired functions and a limited expansion phenotype compared with mild or moderate cases, despite highly expressed proliferation genes [7,8]. The underlying mechanism remains unclear and requires further exploration.

During viral infection, pathogen-specific CD8^+^ T cells are activated, proliferated, and eventually expanded, and the cellular metabolism and mitochondrial function are the main determinants of this process [9,10]. During this period, effector CD8^+^ T cells rely on the enormous energy generated by oxidative phosphorylation and glycolysis to complete the cell cycle [11,12]. The mitochondrial oxidative phosphorylation system is the major energy provider in eukaryotic cells, including five different enzyme complexes (mitochondrial complexes I to V) and two mobile electron carriers. Mitochondrial complexes I (NADH-CoQ reductase), II (succinate-CoQ reductase), III (CoQH2-c reductase), IV (cytochrome c oxidase), and V (oligomycin-sensitive ATPase) catalyze the generation of ATP through a series of redox reactions, and a sufficient energy supply is crucial for the proliferation and expansion of CD8^+^ T cells. Therefore, insufficient cellular metabolism and mitochondrial energy generation may be responsible for the impaired function and limited expansion of CD8^+^ T cells in severe/critical COVID-19.

In this study, through single-cell RNA sequencing (scRNA-seq) in combination with T cell receptor sequencing, a proliferative-exhausted CD8^+^ T cell phenotype with cell cycle arrest and impaired mitochondrial function was observed in severe/critical COVID-19 patients. Our ex vivo experiments further indicated that SARS-CoV-2 ORF3a induced the galectin-3 expression in lung epithelial cells and inhibited the transcription of mitochondrial complex genes and the mitochondrial biogenesis of CD8^+^ T cells, which was responsible for the cell cycle arrest and low expansion of CD8^+^ T cells. Our findings contributed to better understanding the mechanisms of immune dysregulation during severe SARS-CoV-2 infection and provided novel targets for developing COVID-19 treatments.

## 2. Results

### 2.1. CD8^+^ T Cells Largely Decreased in Severe/Critical Patients, Which Is Associated with COVID-19 Progression and Poor Prognosis

**Figure 1:**
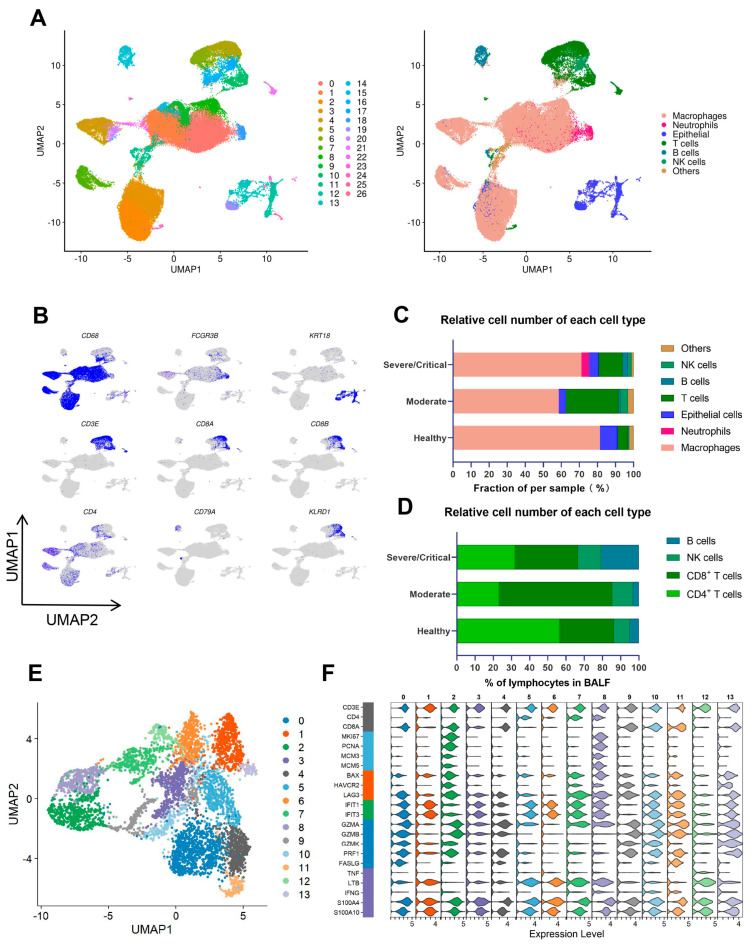
CD8^+^ T cells exhibited a proliferative-exhausted phenotype that correlated with the disease progression of COVID-19. (**A**) Overview of the cell clusters in the integrated single-cell transcriptomes of bronchoalveolar lavage fluid (BALF) cells derived from COVID-19 patients and healthy controls. Uniform Manifold Approximation and Projection (UMAP)of 27 cell clusters and 7 cell types among healthy controls, moderate COVID-19 patients, and severe/critical COVID-19 patients are displayed. (**B**) Marker genes used to identify major cell types were specifically expressed in the corresponding clusters. (**C**) The bar plot compares the proportion of major BALF cell types in healthy controls, patients with moderate COVID-19, and patients with severe/critical COVID-19. (**D**) The bar plot shows the percentage of lymphocyte clusters in healthy controls, patients with moderate COVID-19, and severe/critical COVID-19 patients. (**E**) The UMAP of 14 heterogeneous clusters of T cells isolated from the BALF of COVID-19 patients. (**F**) Violin plots show normalized expression level of representative phenotypic (*CD3E*, *CD4*, *CD8A*), proliferation (*MKI67*, *PCNA*, *MCM3*, *MCM5*), exhaustion (*BAX*, *HAVCR2*, *LAG3*), IFN-induced (*IFIT1*, *IFIT3*), cytotoxic (*GZMA*, *GZMB*, *GZMK*, *PRF1*, *FASLG*), and inflammatory markers (*TNF*, *LTB*, *IFNG*, *S100A4*, *S100A10*), respectively, in T cells from the BALF of COVID-19 patients.

Ever since SARS-CoV-2 first appeared, researchers have been trying to understand how the immune system works during each stage of COVID-19. In this study, we first analyzed the scRNA-seq data of bronchoalveolar lavage fluid (BALF) to measure the T cell responses against SARS-CoV-2 and to investigate the ways by which COVID-19 patients developed into a severe or even critical situation. The Shared Nearest Neighbor (SNN) modular optimization-based clustering algorithm, implemented in Seurat v4, was utilized to identify clusters of BALF cells. Visualization of these identified clusters was achieved by Uniform Manifold Approximation and Projection (UMAP). A total of 27 distinct clusters were shown by clustering analysis, covering diverse cell types in the respiratory system (Figure 1A). Major groups of immune cells in BALF, namely macrophage, neutrophil, T, B, NK, and epithelial cells were identified by combining specific gene expression signatures with the SingleR method [13]. The expression levels of major signatures to annotate these subpopulations are shown in Figure 1B.

The proportion of T cells In the BALF of severe/critical patients was 13.37%, much lower than that of moderate COVID-19 patients (29.33%) (Figure 1C). For the composition of lymphocytes in BALF, CD4^+^ T cells and CD8^+^ T cells in severe/critical patients accounted for 32.04% and 34.63%, while CD4^+^ T cells and CD8^+^ T cells, respectively, accounted for 23.24% and 62.36% in moderate patients (Figure 1D). The proportion of CD8^+^ T cells in severe/critical COVID-19 patients drastically dropped as opposed to moderate patients, which is associated with COVID-19 progression and poor prognosis.

### 2.2. A Proliferative-Exhausted CD8^+^ T Cell Phenotype Was Identified in Severe/Critical COVID-19 Patients through scRNA-Seq and scTCR-Seq Analysis

To further explore the characteristic changes in severe/critical patients, we subset T cells from COVID-19 patients and re-clustered them by unsupervised clustering. The T cell population was divided into 14 distinct clusters (Figure 1E). Cluster 2 expressed *CD8*^+^, cytotoxic signatures (*GZMB* and *PRF1*), and inflammatory proteins (*S100A4* and *S100A10*), and mostly emerged at the stage of severe/critical COVID-19. Meanwhile, cluster 2 displayed high levels of proliferation markers (*MKI67*, *PCNA*, *MCM3*, and *MCM5*) (Figure 1F). The levels of functional genes (*FASLG*, etc.) were also decreased in cluster 2, which revealed that the cellular function of cluster 2 was impaired. Notably, cluster 2 (*CD8*^+^*MCM*^+^*FASLG*^low^ cells) was a group of abnormal CD8^+^ T cells mostly from severe/critical patients and highly expressed proliferative genes, which conflicts with CD8^+^ T cell reduction in severe/critical patients. At the transcriptional level, *MCM*^+^*FASLG*^low^ CD8^+^ T cells were found to highly express exhausted genes such as *HAVCR2* and *LAG3* (Figure 1F).

**Figure 2:**
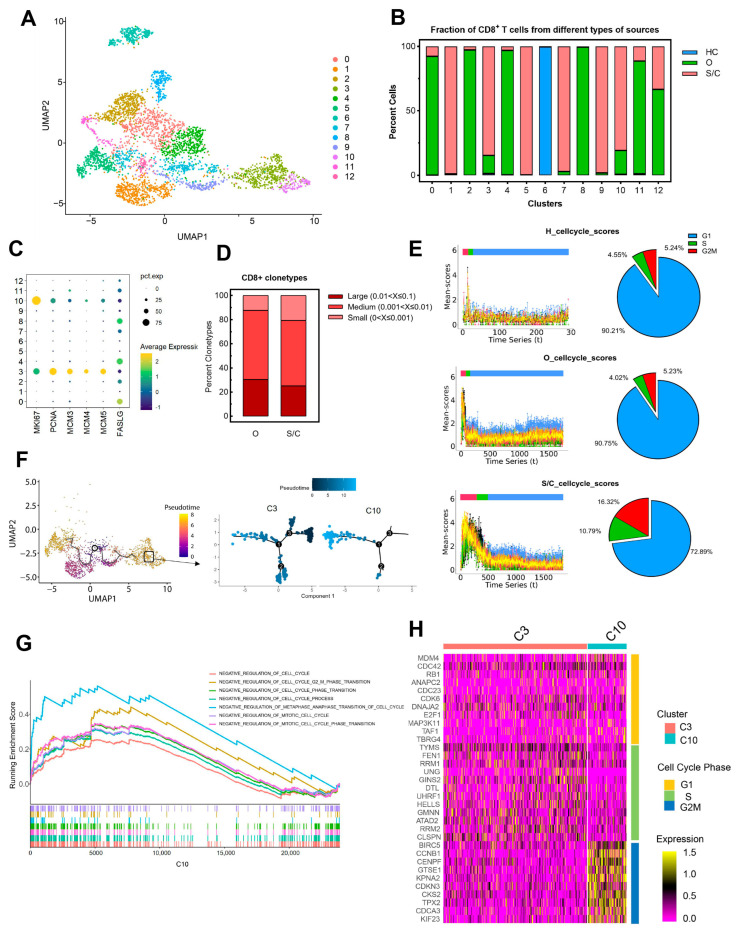
Cell cycle arrest occurred in certain CD8^+^ T cell subpopulations of severe/critical COVID-19 patients. (**A**) UMAP embedding of all CD8^+^ T cells colored by unsupervised clustering. (**B**) Percentage of CD8^+^ T cells across healthy controls, moderate, and severe/critical COVID-19 patients in individual clusters. HC means healthy controls, O means moderate COVID-19 patients, and S/C means severe or critical COVID-19 patients. (**C**) A dot plot showing the expression of proliferation-associated genes and FASLG by CD8^+^ T cell clusters. (**D**) Bar plots show the percentage of clonotypes in specified CD8^+^ T cell clusters from patients with moderate and severe/critical COVID-19. The clonotypes are categorized as Large (0.01 < X ≦ 0.1), Medium (0.001 < X ≦ 0.01), and Small (0 < X ≦ 0.001) based on their relative abundance. (**E**) reCAT reconstructs cell cycle time-series and predicts cell cycle stages along the time-series. The presentation of different cell cycle phases of healthy controls (upper panel), moderate patients (middle panel), and severe/critical patients (lower panel). The corresponding pie charts show the proportion of CD8^+^ T cells at different cell cycle stages. (**F**) Pseudo-time trajectory projected onto a UMAP of CD8^+^ T cells from severe/critical COVID-19 patients. Pseudo-time values are color-coded. Numbers in circles indicate inferred differentiation paths. The enlarged box is the timing diagram of C3 and C10. C3, C10: two CD8^+^ T cell groups isolated from severe/critical COVID-19 patients. (**G**) Enrichment plots for pathways identified by GSEA. C10 was positively correlated with the negative regulation of the cell cycle relative signaling pathway in the Molecular Signatures Database (MSigDB). (**H**) Heatmap showing average expression of cell cycle phases’ gene signatures (rows) in C3 and C10 (columns). The color scale shows the expression level of selected transcripts in each cell.

We then selected all of the CD8^+^ T cells from the BALF cells to further interrogate the key role of *MCM*^+^*FASLG*^low^ CD8^+^ T cells in severe/critical COVID-19 patients. The characteristic signatures of CD8^+^ T cells and other cells were identified, and the varying degree of the expression of signatures in specific cell clusters was visualized. Finally, 3870 CD8^+^ T cells displayed signatures of CD3 and CD8, and were isolated from BALF cells to perform subsequent analysis. These CD8^+^ T cells were clustered into 13 subpopulations and were clearly differentiated according to their sample origin (Figure 2A,B). The proportions of CD8^+^ T cells from people with/without COVID-19 are shown in each cell cluster (Figure 2B). These clusters showed a distinguishing pattern in the low-dimensional distribution. Cluster 6 consisted of the cells of healthy persons, and was distinct from other clusters of cells from COVID-19 patients, which revealed that CD8^+^ T cells from COVID-19 patients and healthy people have obvious heterogeneity (Figure 2B). Cluster 3 and cluster 10 were from COVID-19 patients, and highly expressed proliferative genes (Figure 2C).

In order to examine the extent of their expansion, the clonotypes were further analyzed. Consistent with the decline in the proportion of CD8^+^ T cells, the clonotype size of cluster 3 and cluster 10 (*MCM*^+^*FASLG*^low^ CD8^+^ T cells) from severe/critical patients was much lower than that of corresponding moderate patients (Figure 2D). These findings suggest that CD8^+^ T cell cloning is actually hindered, and a proliferative-exhausted CD8^+^ T cell phenotype is presented in severe/critical COVID-19 patients.

### 2.3. Critical CD8^+^ T Cell Subpopulations Have Cell Cycle Arrest and Are Correlated with the Disease Progression of COVID-19

To further investigate the contradiction behind this, we analyzed the cell cycle of these CD8^+^ T cell subpopulations with reCAT [14]. The cell cycle is known to have wide-ranging effects on cellular physiology and can modulate differentiation and gene expression profiles. Proliferation depends on the smooth completion of four distinct phases of the cell cycle: G0/G1, S, G2, and M [15]. As shown in Figure 2E, severe/critical COVID-19 patients had significantly more CD8^+^ T cells in the G2/M phase than that in moderate patients and healthy people. Among the CD8^+^ T cells of severe/critical COVID-19 patients, 10.79% were in the S phase and 16.32% were in the G2/M phase. In contrast, the cell cycle distributions of moderate patients and healthy people were similar, with 4.02% or 4.55% in the S phase and 5.23% or 5.24% in the G2/M phase (Figure 2E). These results indicated that the CD8^+^ T cells from severe/critical COVID-19 patients had cell cycle arrest.

MKI67, which is associated with cellular proliferation, was only expressed in cluster 3 and cluster 10. In the meanwhile, we also found that *PCNA*, *MCM3*, *MCM4*, and *MCM5*, which are essential for the initiation of eukaryotic genome replication, were highly expressed in cluster 3 (Figure 2C). Differences in the expression levels of the proliferation genes between cluster 3 and cluster 10 may imply their different roles in severe/critical patients. C3 and C10 represent cluster 3 and cluster 10, respectively, which were cells from severe/critical COVID-19 patients. Pseudo-time analysis revealed a differentiation trajectory of CD8^+^ T cells from severe/critical COVID-19 patients, which showed that C10 was at the rear end of C3 (Figure 2F). Collectively, these results raised the possibility that C10 was developed from C3.

GSEA analysis showed that numerous signaling pathways of negative regulation were involved in the cell cycle of C10 (Figure 2G). To be more precise, we next checked the expression of cell-cycle-related genes, and found that C10 mainly expressed the G2/M phase genes, such as *BIRC5*, *CCNB1*, *CENPF*, *KPNA2*, and *CKS2* (Figure 2H). Taken together, C10 was a cluster of cells that could not complete expansion due to cell cycle arrest at the G2/M phase, which were cells from severe/critical patients. These findings explained a potential reason why CD8^+^ T clonotypes were not enlarged in severe/critical cases.

### 2.4. Impairment of Mitochondrial Function in the Cell-Cycle-Arrest Cluster

**Figure 3:**
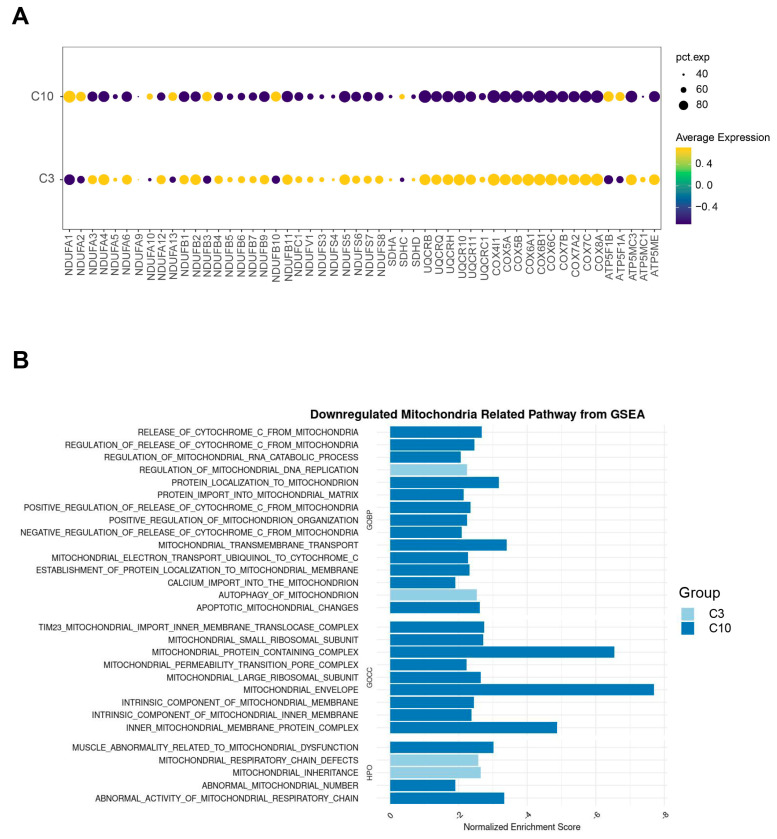
Impaired mitochondrial function in CD8^+^ T cells from severe/critical COVID-19 patients. (**A**) The expression of mitochondrial complex-related genes in different cell subsets (C3 and C10) is depicted by dot plot. Pct. exp indicates the percentage of cells expressing the gene. Color scale shows the average expression level of mitochondrial complex-related genes. (**B**) Gene Set Enrichment Analysis (GSEA) for specific CD8^+^ T cells in severe/critical COVID-19 patients on downregulated mitochondria-related pathways. Column chart showing the normalized enrichment scores for the mitochondria-related pathways derived from BP, CC, and HPO that are significantly downregulated in C3 or C10.

T cell proliferation and activation require ATP produced by mitochondrial activity [16]. To further explore the mechanism of the cell cycle arrest of C10, we investigated the expression of the mitochondrial respiratory chain complex-related gene expression of C3 and C10. C3 highly expressed *NDUFA4*, *NDUFB2*, *NDUFB11*, *NUDFS5* (encoding mitochondrial respiratory chain complex I), *UQCRB*, *UQCRQ*, *UQCRH*, *UQCR10* (encoding complex III), *COX4I1*, *COX6A1*, *COX6C*, and *COX7A2* (encoding complex IV), but C10 had very low relative expression of these genes (Figure 3A). We next collected mitochondria-related signaling pathways from the MSigDB database and separately performed GSEA analysis of C3 and C10. Enrichment results of C10 showed plenty of downregulation of mitochondria-related pathways, while enriched terms in C3 had little relationship with mitochondrial function (Figure 3B). Strong downregulation of respiratory chain-related genes and mitochondria-related signaling pathways resulted in the suppression of mitochondrial biogenesis and function impairment in C10, and thereby reduced energy availability for cellular processes.

### 2.5. Significant Galectin-Associated Interactions between Lung Epithelial Cells and Abnormal CD8^+^ T Cells in Severe/Critical COVID-19 Patients

**Figure 4:**
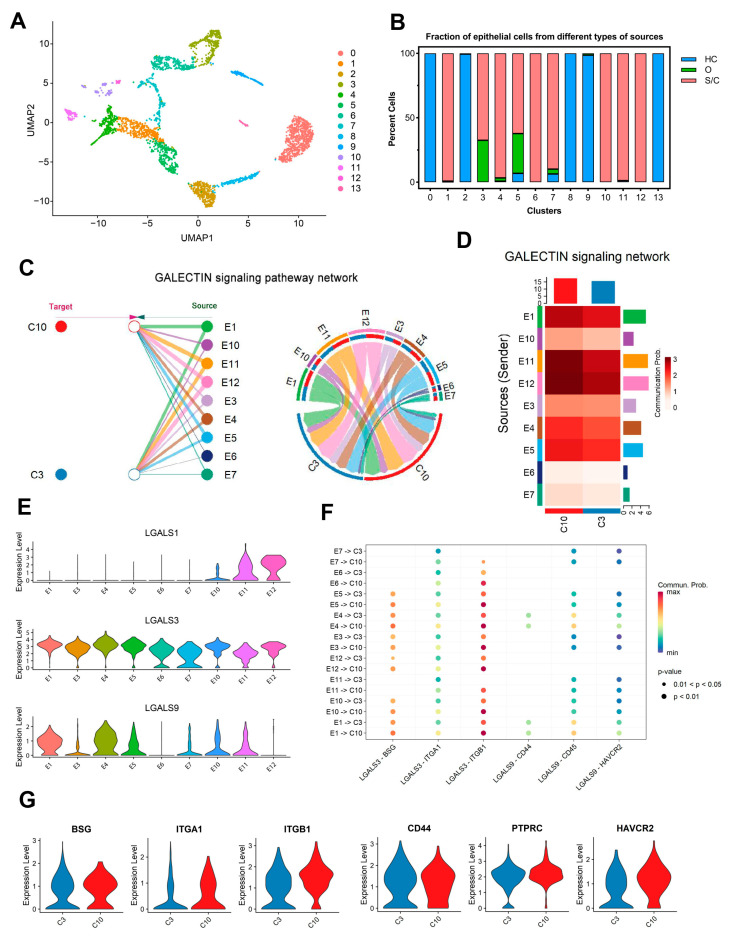
Galectin signaling pathways may be involved in the mitochondrial dysfunction of CD8^+^ T cells in severe/critical COVID-19 patients. (**A**) The UMAP presentation of 14 heterogeneous clusters of epithelial cells. (**B**) Percentage of epithelial cells across healthy controls (HC, blue), moderate (O, green), and severe/critical (S/C, red) COVID-19 patients in individual clusters. (**C**) Hierarchical plot (left) and chord diagram (right) show the inferred intercellular communication network (only the effect of epithelial cells on CD8^+^ T cells is represented here) for galectin signaling. E1, 3–7, 10–12: nine epithelial cell groups separated from severe/critical COVID-19 patients; C3, 10: two CD8^+^ T cell groups from severe/critical COVID-19 patients. Different colors represent different cell clusters. (**D**) CellChat infers the strength of different cell groups as senders or receivers of signals during cellular communication. The color bar shows the strength of signals, the histograms with different colors indicate the total strength of different cell groups, and the x- and y-axes represent the signal receiver (CD8^+^ T cells) or sender (epithelial cells). (**E**) Violin plots comparing the normalized expression level of *LGALS1*, *LAGLS3*, and *LGALS9* transcripts in various epithelial clusters of COVID-19 patients with severe/critical disease. (**F**) Bubble plot showing the stronger effects of multiple galectin ligand–receptor pairs on C10 involved in cellular communication. The color bar shows the strength of signals. (**G**) Violin plots exhibit the expression levels of relative receptor genes in C3 and C10.

The function and phenotype of immune cells are largely influenced by the environment, and the target cells of immune responses are usually involved in reshaping the immune environment and regulating immune cells [17]. We re-clustered six subclusters of the lung epithelial cells of BALF to further dissect their heterogeneity and to explore their different effects on C3 and C10 of CD8^+^ T cells. Ultimately, the re-screened epithelial cell population was divided into 14 subpopulations (Figure 4A). There was a total of 3338 epithelial cells, and their distribution in each cell population was graphed in Figure 4B. For the convenience of description, we abbreviated the epithelial subpopulations derived from severe/critical COVID-19 patients as E1, E3, E4, E5, E6, E7, E10, E11, and E12. We chose lung epithelial cells derived from severe/critical COVID-19 patients to conduct cell communication analysis with C3 and C10. The R toolkit CellChat (v1.5.0) for single-cell data was applied to analyze the signaling pathways that are possibly involved in epithelial–CD8^+^ T cell interactions. The galectin signaling pathway was found especially prominent among the secreted signaling pathways (Figure 4C). Galectins are a family of β-galactoside-binding proteins, which are known for their pro-adhesive potential and their negative effects on T cell proliferation and survival [18].

The CellChat official database only contains three ligand–receptor pairs involved in the galectin signaling pathway. In order to deeply investigate the role of galectin-related interactions between epithelial cells and CD8^+^ T cells from severe/critical COVID-19 patients, an extra ligand–receptor interaction list, containing more than one hundred ligand–receptor pairs, has been supplemented according to official instructions (Table S1). In the galectin signaling pathways, all ligand–receptor interactions were computed by CellChat. E1, E4, and E5, the three subpopulations with a higher cell count, had a significant effect on C3 and C10 through the galectin signaling pathways (Figure 4D). Moreover, for each epithelial cell cluster, the strength of their interaction with C10 was higher than that with C3 (Figure 4C,D).

*LGALS3* and *LGALS9,* encoding galectin-3 and galectin-9, respectively, were highly expressed in E1 and E4 (Figure 4E). Ligand–receptor analysis of *LGALS3* and *LGALS9* found that there were six ligand–receptor interactions, named *LGALS3*-*BSG*, *LGALS3*-*ITGA1*, *LGALS3*-*ITGB1*, *LGALS9*-*CD44*, *LGALS9*-*CD45*, and *LGALS9*-*HAVCR2*, which showed that E1 and E4 had a stronger effect on C10 than C3 (Figure 4F). Moreover, the receptors (*ITGB1*, *PTPRC*, and *HAVCR2*) were upregulated in C10 compared to C3 (Figure 4G). *LGALS3* or *LGALS9* bound to these receptors, and downregulated CD8^+^ T proliferation and cell function in severe/critical COVID-19 patients.

### 2.6. SARS-CoV-2 ORF3a Induces High Expression of Epithelial Galectin-3, and Inhibited Mitochondrial Complex-Related Gene Expression and Biogenesis of CD8^+^ T Cells

ORF3a is an accessory protein encoded by SARS-CoV-2, which not only facilitates viral release, but has also been shown to affect a variety of physiological processes in host cells, including inducing apoptosis, blocking autolysosome formation, and triggering inflammatory responses [19,20]. Thus, we wondered if SARS-CoV-2 ORF3a (referred to as ORF3a hereafter) increased galectin expression in epithelial cells, and mediated the downstream changes in CD8^+^ T cells.

**Figure 5:**
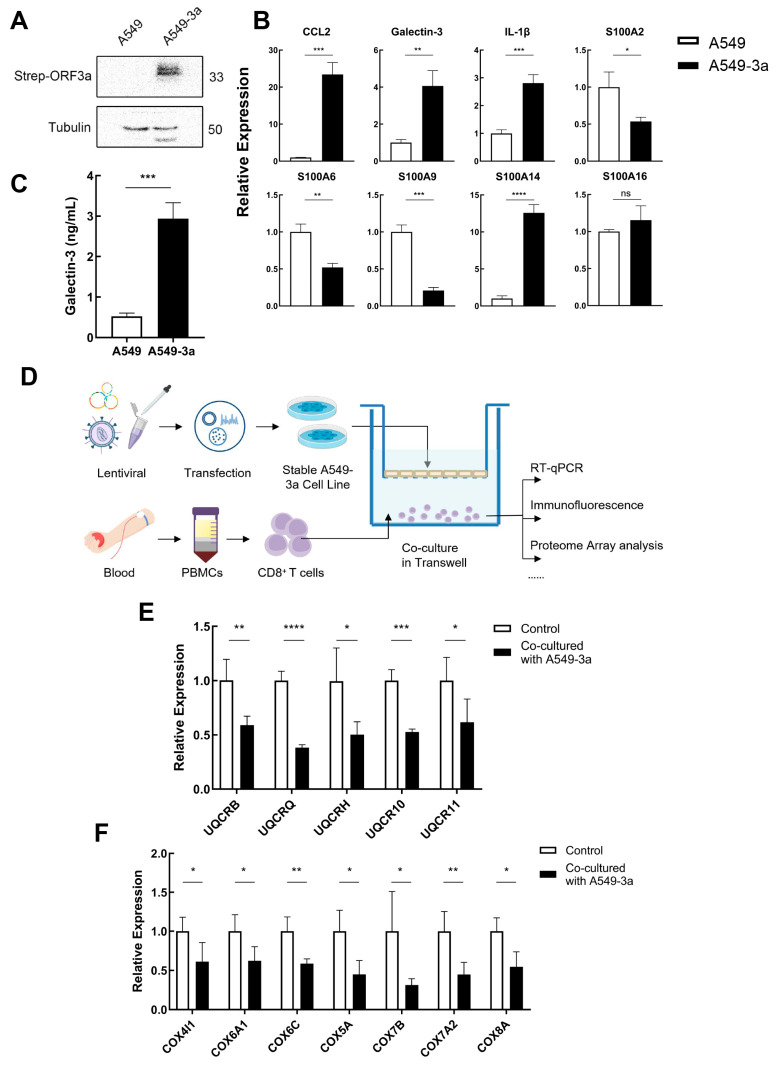
SARS-CoV-2 ORF3a induced galectin-3 expression in A549 cells and impaired mitochondrial biogenesis of CD8^+^ T cells. (**A**) Representative Western blot images showing the expression of SARS-CoV-2 ORF3a in A549-3a cells (A549 cells transfected with ORF3a). (**B**) Inflammatory *S100*, *IL-1β*, *galectin-3*, and *CCL2* mRNA levels in A549/A549-3a cells were assessed by real-time PCR. Relative gene expression was normalized against *GAPDH*. (**C**) Galectin-3 expression level in supernatant was detected by ELISA. (**D**) Experimental design to characterize the alternation of CD8^+^ T cells after co-culture with epithelial cells expressing ORF3a. CD8^+^ T cells were isolated from the PBMC of healthy donors to co-culture with A549/A549-3a cells for 48 h, and then subjected to multiple assays. (**E**,**F**) The mRNA expression levels of ETC complexes III (**E**) and IV (**F**) in CD8^+^ T cells after co-culture with A549/A549-3a cells were determined by real-time PCR. * *p* < 0.05, ** *p* < 0.01, *** *p* < 0.001, **** *p* < 0.0001, ns stands for not significant.

To verify the assumption, human lung epithelial cells (A549 cells) were infected with SARS-CoV-2 ORF3a lentivirus, and a stable cell line (termed A549-3a) was generated after puromycin selection. The production of the ORF3a protein in A549-3a cells was subsequently detected and confirmed by Western blot (Figure 5A). Next, the role of ORF3a in regulating pro-inflammatory cytokine production was evaluated. The results showed that *galectin-3*, *S100A14*, *IL-1β*, and *CCL2* mRNAs were significantly induced upon ORF3a transfection into A549 cells (Figure 5B). Moreover, ELISA confirmed a significant increase in the secretion of galectin-3 (Figure 5C). These results were in line with the previous report that ORF3a caused inflammation, and confirmed our conjecture that SARS-CoV-2 ORF3a induces high levels of galectin-3 in lung epithelial cells.

To study the interaction between lung epithelial cells and CD8^+^ T cells ex vivo, we designed the A549-3a/CD8^+^ T cell co-culture system and the experimental procedure is shown in Figure 5D. After 36 h of co-culture with lung epithelial cells under anti-CD3/anti-CD28 stimulation, CD8^+^ T cells were harvested and detected. The qPCR results showed that after co-culture with A549-3a, the gene expression levels of the mitochondrial complex III/IV of CD8^+^ T cells were significantly reduced (Figure 5E,F). Among them, the expression of *UQCRQ*, *UQCR10*, *UQCRB*, *COX6C*, and *COX7A2* were highly significantly decreased compared with the control group (*p* < 0.01). This suggested that lung epithelial cells infected with SARS-CoV-2 ORF3a could downregulate mitochondrial complex-related gene expression and impair mitochondrial biogenesis in CD8^+^ T cells.

### 2.7. Galectin-3 Signaling Downregulated Mitochondrial Complex III/IV Genes Transcription and Biogenesis by NRF-1 Suppression

**Figure 6:**
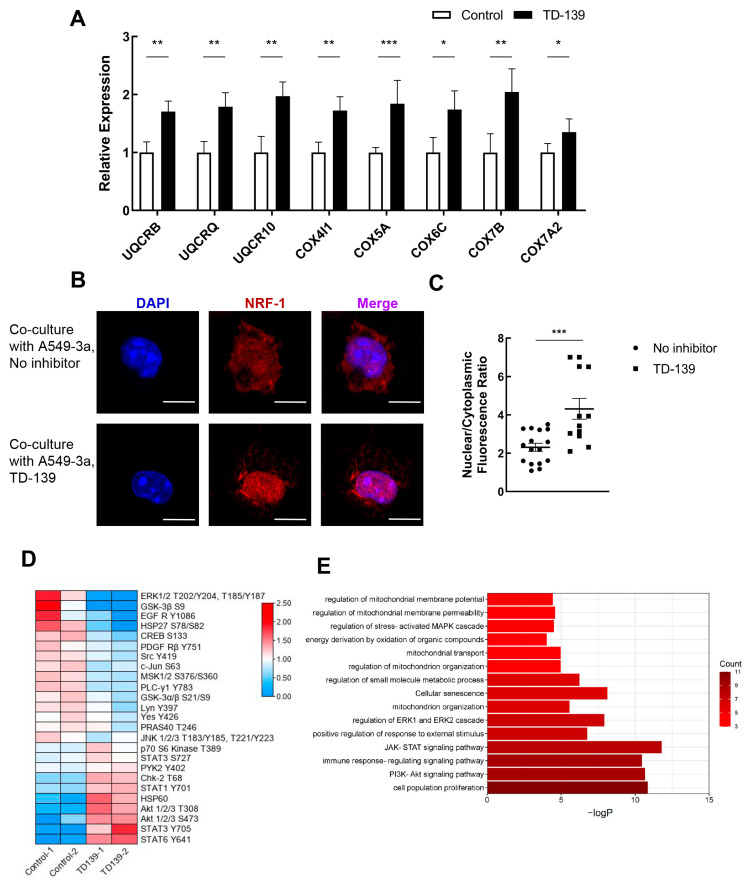
Galectin-3 inhibited the nuclear translocation of NRF-1 and the transcription of ETC III/IV genes through ERK- and Akt-related signaling pathways. (**A**) The expression of ETC complexes III and IV genes in CD8^+^ T cells co-cultured with A549-3a cells. The TD-139 group was treated with the galectin-3 inhibitor TD-139 for 48 h. (**B**) CD8^+^ T cells were examined by confocal microscopy for the localization and expression of NRF-1 (red). Nuclei were visualized using DAPI counterstain (blue). All scale bars correspond to 5 µm. (**C**) Quantification of nuclear fluorescence intensities of NRF-1. (**D**) Phosphokinase production of CD8^+^ T cells co-cultured with A549-3a cells for 24 h in the presence or absence of TD-139 was compared using a commercial protein array kit. After quantification, 25 differentially expressed proteins were identified and visualized in heatmap. The color bar on the left represents the relative expression levels. (**E**) Bar diagram of the enrichment analysis of differentially expressed proteins involved in (**D**). Color indicates the number of genes involved in a specific signaling pathway. * *p* < 0.05, ** *p* < 0.01, *** *p* < 0.001.

Our single-cell transcriptome analysis revealed that epithelial galectin-3 and receptor interactions were associated with the impaired mitochondrial function and cell cycle arrest of CD8^+^ T cells in severe/critical COVID-19. To investigate the role of galectin-3 in the impaired mitochondrial biogenesis of CD8^+^ T cells in COVID-19 patients, TD-139, a high-affinity inhibitor of galectin-3, was utilized in the A549-3a/CD8^+^ T cell co-culture system. As speculated, the transcriptional repression of mitochondrial complex III/IV genes (*UQCRB*, *UQCRQ*, *UQCR10*, *COX4I1*, *COX5A*, *COX6C*, *COX7B*, and *COX7A2*) was differentially rescued with TD-139 treatment (Figure 6A).

In order to meet the energy and metabolic needs of cell proliferation, the number and function of mitochondria are finely regulated, which is largely accomplished at the transcriptional level. Nuclear respiratory factor 1 (NRF1), a transcription factor involved in a wide range of gene expressions and mitochondrial biogenesis, has received considerable attention in recent years [21,22,23]. Here, immunofluorescence analysis showed that, in the co-culture system treated with TD-139, the fluorescence intensity of NRF1 in the nuclei of CD8^+^ T cells was strongly increased compared with the non-inhibitor group (Figure 6B,C). Taken together, our findings suggest that galectin-3 mediates the downregulation of mitochondrial complex III/IV genes by suppressing NRF1 nuclear translocation and transcriptional activation.

### 2.8. ERK and Akt Signaling Pathways Were Involved in CD8^+^ T Cell Mitochondrial Dysfunction

To determine which signaling cascade galectin-3 functions through, the phosphorylation levels of kinases involved in signaling pathways were examined using the proteome profiler array. After co-culture with or without inhibitor treatment, CD8^+^ T cells in the lower chamber were collected and proteins were extracted and then examined. Quantification and statistical analysis of the results identified 25 differential proteins in CD8^+^ T cells, and ERK, GSK-3, and EGFR were the most significant downregulated proteins in the inhibitor-treated group (Figure 6D). The markedly increased proteins included multiple transcription factors (STAT family members), protein kinase B (Akt), and the molecular chaperone HSP60 (Figure 6D). It is interesting to note, the upregulation of Akt signaling and HSP60 is consistent with their roles in promoting mitochondrial homeostasis. Furthermore, functional enrichment analysis showed that differential proteins were enriched in signaling pathways, including the regulation of the mitochondrial membrane and mitochondrion organization (Figure 6E). Our results showed that the downstream effect of galectin-3 was correlated with ERK activation and Akt suppression, which can be reversed by inhibitor treatment.

## 3. Discussion

Some studies have shown that the decrease and depletion of CD8^+^ T cells are negatively correlated with the prognosis of COVID-19, especially in patients requiring intensive care [24,25,26,27], but its mechanisms were not fully understood. Understanding SARS-CoV-2-specific CD8^+^ T cell responses is critical for developing effective strategies to fight against the virus and prevent adverse clinical outcomes. In this study, we utilized computational methods to integrate and analyze the single-cell characteristics of BALF, combined with ex vivo experiments to explore the ways by which CD8^+^ T cells are dramatically decreased and depleted in patients with severe/critical COVID-19.

Naive CD8^+^ T cells will undergo proliferation, clonal expansion, and differentiation into effector CD8^+^ T cells during virus infection [10]. Our single-cell analysis showed that, in patients with severe/critical COVID-19, CD8^+^ T cell clusters were found to have a proliferative phenotype with highly expressed *MKI67* and *PCNA*. However, the proportion of CD8^+^ T cells in severe/critical patients decreased significantly compared to that of moderate patients, which suggested ineffective or failed clonal expansion in severe/critical patients. Additionally, we observed that CD8^+^ T cells in this group had partially impaired immune functions. We analyzed the intercellular heterogeneity of this cluster and divided it into two subgroups (named C3 and C10).

TCR immune repertoire analysis also revealed that these two CD8^+^ T cell clusters in severe/critical COVID-19 patients were less clonal than those in corresponding clusters in moderate patients, which indicated that these cells did not effectively proliferate to effector CD8^+^ T cells as they should. Moreover, CD8^+^ T cells derived from severe/critical patients showed an increased proportion of cells in the S and G2/M phases, which suggested a blockade of the cell cycle process. The pseudo-time analysis also demonstrated that these two populations were at the end of the developmental trajectory, implying that these “proliferative-exhausted” cells may represent a divergent destination for naive CD8^+^ T cells. Although C10 and C3 shared the same proliferative phenotype, C10 exhibited an obvious G2/M blockade with a high level of G2/M gene expression. Gene Set Enrichment Analysis (GSEA) revealed that several signaling pathways were involved in the negative regulation of the cell cycle in C10. These results suggested that these two CD8^+^ T cell clusters could be regulated differently and lead to different transcriptional activities.

Energy metabolism plays a crucial role in the proliferation and expansion of immune cells. Upon stimulation, CD8^+^ T cells rapidly change their metabolic energy supply from a resting state to increased mitochondrial metabolism [28]. Our single-cell analysis and experimental results confirmed that the CD8^+^ T cells with cell cycle arrest had impaired mitochondrial biogenesis. Genes encoding subunits of electron transport chain (ETC) complexes were downregulated at the transcriptional level, leading to an insufficient energy supply to meet the consumption required for cell expansion. This could explain why the specific CD8^+^ T cell subsets in severe/critical patients expressed proliferation-related signatures, but ultimately did not lead to T cell expansion: they could not complete mitosis.

Previous studies have reported that galectin-3 upregulation can serve as a prognostic biomarker in COVID-19 patients [29,30], but its role in the disease process was not entirely clear. SARS-CoV-2-infected epithelial cells trigger senescence-like cell cycle arrest in neighboring uninfected cells in a paracrine manner via virus-induced cytokine production [31,32]. Our cell communication analysis indicated that lung epithelial cells were involved in the cell cycle arrest of CD8^+^ T cells. Galectin-3, a member of the β-galactoside-binding lectin family, was highly expressed by epithelial cells and had a strong effect on C10 of CD8^+^ T cells in patients with severe/critical COVID-19. ORF3a, an accessory protein of SARS-CoV-2, plays an important role in the immunopathogenesis of COVID-19. We transfected the lung epithelial cells with ORF3a (named A549-3a), and co-cultured A549-3a with CD8^+^ T cells. The ex vivo studies further verified that SARS-CoV-2 ORF3a expression upregulated various pro-inflammatory factors and proteins, including galectin-3 in A549-3a, and epithelial galectin-3 signaling downregulated mitochondrial biogenesis and promoted the G2/M cell cycle arrest of CD8^+^ T cells.

NRF1 is a nuclear respiratory factor that initiates the transcription of multiple mitochondrial respiratory chain-related genes [33,34]. When CD8^+^ T cells were co-cultured with lung epithelial cells transfected with ORF3a, NRF1 activation and nuclear translocation were inhibited, and the expression of mitochondrial complex genes was downregulated in CD8^+^ T cells. In the co-culture system, TD-139, a galectin-3 inhibitor, promoted the NRF1 activation and nuclear translocation, and restored the expression of mitochondrial complex genes. Other galectin-3 inhibitors have also been reported to ameliorate mitochondrial damage in the hearts of obese rats [35]. These findings raise the possibility of developing galectin-3 inhibitors and NRF1 activators as potential treatment options for COVID-19 patients.

Our comparative analysis of galectin-3 inhibitor-treated and untreated groups revealed multiple changes in different kinases, particularly in ERK- and Akt-related signaling pathways. ERK 1/2 is a master of transduction signals from surface receptors to the nucleus. In tumors and neurodegenerative diseases, ERK 1/2 inhibited mitochondrial biogenesis by phosphorylating different effector molecules [36,37]. Consistent with previous studies, we found that the galectin-3 inhibitor significantly reduced ERK phosphorylation in CD8^+^ T cells, which was accompanied by promoting the transcription of mitochondrial complex genes. In contrast, Akt phosphorylation was upregulated in the galectin inhibitor-treated group. Akt phosphorylation promoted the activation of PGC-1α, an important regulator of mitochondrial biogenesis that enhances respiratory capacity and maintains homeostasis [38]. The regulation of these two kinases upregulated the transcription of mitochondrial complex genes and improved mitochondrial biogenesis. Additionally, the galectin-3 inhibitor upregulated the transcription factors of the STATs family, indicating the restoration of the CD8^+^ T lymphocyte response to interferon signals and antiviral function.

In conclusion, our study identified a specific CD8^+^ T cell subset in severe/critical COVID-19 patients, which was characterized by abnormally high proliferative gene expression and cell cycle arrest. Galectin-3 produced by epithelial cells with a high expression of SARS-CoV-2 ORF3a inhibited the transcription of mitochondrial respiratory chain complex genes and resulted in mitochondrial biogenesis impairment and insufficient energy supply, eventually preventing the processes of CD8^+^ T cell proliferation and expansion. Blocking galectin-3 could be an effective solution to relieve the cell cycle arrest of CD8^+^ T cells in COVID-19 patients and restore their expansion ability and cell function. TD-139, a galectin-3 inhibitor, has been already available as a treatment for idiopathic pulmonary fibrosis (IPF) [39]. Our study provides new insight into the benefits of galectin-3 suppression in COVID-19 patients, which could save the lives of severe/critical patients, and also improve the lung function of patients who survive severe/critical COVID-19.

## 4. Materials and Methods

### 4.1. Research Sources

HEK293T cell line and A549 cell line in our study were obtained from the American Type Culture Collection (ATCC, Garfield, NJ, USA). The pLVX-EF1alpha-SARS-CoV-2-orf3a-2xStrep-IRES-Puro plasmid was constructed by Nevan Krogan lab, and provided by Addgene, Watertown, MA, USA. Lentiviral particles were packaged by the 2nd Generation Packaging System, in which the packaging plasmid GAG and the envelope plasmid VSV-G were obtained from Addgene, USA.

We obtained the single-cell RNA sequencing and TCR sequencing data of COVID-19 from GEO; the accession numbers were GSE145926 and GSE128033. These datasets provided single-cell sequencing data of BALF from four healthy subjects (HC1–HC4), three moderate COVID-19 patients (M1–M3), and six severe/critical infection patients (S1–S6), in which BALF samples from COVID-19 patients also underwent TCR sequencing [6,40]. These COVID-19 patients were all admitted and sampled in January 2020.

### 4.2. The Data Process and Analysis of Single-Cell RNA-Sequencing and TCR Sequencing

We performed subsequent calculations on the count matrices using the R package Seurat (https://satijalab.org/seurat/, accessed on 5 May 2022). First, the cells were filtered based on the standard that the number of expressed genes was less than 200 or greater than 6000, and the percentage of mitochondrial genes was greater than 0.2. In addition, the filtered expression matrix was normalized using the ‘LogNormalize’ method and the scaling factor was set to 10,000. Using the “vst” method in the Seurat function “FindVariableFeatures”, the top 2000 variable features were identified. The expression values of all genes were transformed by z-score using the “ScaleData” function to obey the normal distribution. Then, principal component analysis (PCA) was performed through the top 2000 variable genes, and the data were dimensionality reduced according to the first 20 principal components. Next, based on the PCA-reduced data, the “FindClusters” function was used for cluster analysis, and the resolution was set to 0.6. Finally, the Unified Manifold Approximation and Projection (UMAP) analysis was performed using the first 20 principal components for visualization.

We initially annotated the cell clusters based on canonical cell markers as follows: B cells (*CD79A*), CD4^+^ T cells (*CD3E* and *CD4*), CD8^+^ T cells (*CD3E*, *CD8A* and *CD8B*), NK cells (*KLRD1*, *FCGR3A* and *NCAM1*), macrophages (*CD68*, *CST3* and *FCGR3A*), neutrophils (*FCGR3B*), and epithelial cells (*KRT18*). To ensure the accuracy of the cell-type assignments, the SingleR algorithm (https://github.com/dviraran/SingleR, accessed on 7 May 2022) was also performed independently. For single-cell immune repertoire data analysis, we utilized the scRepertoire package (v1.0.2) combined with the Seurat package to display the clonotype distribution of clusters defined by the scRNA-seq data.

Cell differentiation trajectories were reconstructed using the pseudo-temporal inference algorithm Monocle 3 (https://github.com/cole-trapnell-lab/monocle-release/, accessed on 21 June 2022). Specifically, we focused on CD8^+^ T cells and identified different branches in the cell trajectory that distinguished various cell states. By considering relevant biological background, we determined the “root” starting point and inferred the order of cell population differentiation. Additionally, we employed Monocle 2 to further investigate the cluster and lineage relationships of two specific cell clusters in CD8^+^ T cells. To investigate the cell cycle arrest of T cells, we utilized the R package reCAT (https://github.com/tinglab/reCAT, accessed on 20 June 2022) and applied default and recommended parameters for the pipeline.

We performed gene set enrichment analysis (GSEA) using the R package fgsea and the C5, the Ontology Gene Sets from the MsigDB database (https://www.gsea-msigdb.org/gsea/msigdb, accessed on 6 July 2022). The fgsea results were initially screened against the criterion of adjusted *p*-value < 0.05. For specific analysis, the downregulated mitochondria-related pathways from the GSEA table were selected based on the normalized enrichment score < 0. In addition, the comparison results of related clusters were visualized via ggplot2 (v3.4.1).

CellChat v1.5.0 (https://github.com/sqjin/CellChat, accessed on 10 July 2022) was performed to evaluate cell-cell interactions according to the expression of known ligand–receptor pairs in different cell types. Specifically, we added more than 100 related ligand–receptor pairs following official guidance from CellChat for further analysis of the galectin pathways. The results were visualized and displayed by relevant functions within the CellChat package.

### 4.3. Generation of Stable Cell Line Expressing SARS-CoV-2 ORF3a

To construct stable cell lines, 293T packaging cells were used for lentiviral packaging and amplification. A total of 293T cells were seeded in 6-well plates and reached 60–70% confluency the next day. The premixed plasmid and PEI (MW25000Da, Sigma, St. Louis, MO, USA) were transfected into 293T cells and incubated for 48 h. Then we collected the supernatant containing lentiviral particles and filtered it through 0.22 µm filters. Lentiviral particles infected A549 cells with polybrene (Sigma, USA) at a final concentration of 8 μg/mL. The A549 cells expressing SARS-CoV-2 ORF3a (A549-3a in abbreviation) were selected with puromycin, and ORF3a expression in A549 was verified by Western blot assay.

### 4.4. RNA Extraction and Real-Time PCR

We extracted RNA from cell samples using the RNAsimple RNA Kit (Tiangen, Beijing, China) following the manufacturer’s instructions. The extracted RNA was subsequently reverse transcribed into cDNA by ABSScript II RT Mix (ABclonal, Wuhan, China). Real-time PCR was performed using 2 × Universal SYBR Green kit (ABclonal, Wuhan, China), and fluorescent signals were collected by CFX96 PCR instrument (Bio-Rad, Hercules, CA, USA). Three runs were performed for each sample. The results were analyzed and examined as the relative mRNA levels based on cycle threshold (CT) values, which were converted to fold changes. The primers were designed using Primer-blast, NCBI (www.ncbi.nlm.nih.gov, accessed on 27 July 2022).

### 4.5. Western Blot

Stable cell lines were harvested and lysed with RIPA lysis buffer (Servicebio, Wuhan, China) for 20 min on ice. Protein concentration was determined using the BCA protein concentration assay kit (Biosharp, Hefei, China). Equal amounts of proteins (2 μg/μL) were loaded onto 10% gels and separated by SDS-PAGE. Subsequently, they were electrophoretically transferred to PVDF membranes. Non-specific areas on the PVDF membrane were blocked by 5% non-fat milk in PBST for 1 h. Next, we incubated the membranes with primary antibodies (β-tubulin, Strep II-Tag, Abclonal, Wuhan, China) overnight at 4 °C. We then incubated them with HRP-coupled goat anti-rabbit IgG secondary antibody for 1 h at room temperature. Finally, the protein bands were visualized using an ECL kit (Meilunbio, Dalian, China) and imaged on ChemiDoc XRS+ (Bio-Rad, USA).

### 4.6. CD8^+^ T Cells Isolated and Co-Cultured with A549-3a

The 24-well plates were coated with CD3 antibody (OKT3, Invitrogen, Carlsbad, CA, USA) overnight. PBMCs were isolated from the peripheral blood of healthy donors using Human PBMC Isolate kit (TBD, Tianjin, China). CD8^+^ T cells were positive selected from PBMC using magnetic beads coated with CD8 antibodies (BD, Franklin Lakes, NJ, USA). These CD8^+^ T cells were placed into the Transwell lower chamber along with CD28 antibody (Invitrogen, USA) and IL-2 (Procell, Wuhan, China). A549 or A549-3a cells were seeded in Transwell inserts (Corning, Corning, NY, USA). For certain experiments, cells were treated with the galactin-3 inhibitor (TD-139) (Selleck, Houston, TX, USA). The co-culture system was incubated for 48 h at 37 °C and 5% CO_2_.

### 4.7. Phosphokinase Chip Array

To explore important signaling in CD8^+^ T cells, we used a human phosphokinase array kit (ARY003C, R&D Systems, Minneapolis, MN, USA) following the manufacturer’s protocol. Firstly, cell lysates were prepared and incubated on membranes containing human phosphokinase antibodies overnight at 4 °C. Then, detection antibody cocktail and membranes were incubated at 4 °C. After washing, HRP-conjugated secondary antibody cocktail was incubated on membranes. Chemi Reagent Mix was subsequently added on membranes, and dot blots were visualized via chemiluminescence (Bio-rad, Hercules, CA, USA). Finally, we used Fiji software (https://imagej.net/Fiji, accessed on 20 August 2022) to quantify spot signals.

### 4.8. Immunofluorescence Staining and Laser Scanning Confocal Microscope Analysis

CD8^+^ T cells were seeded on climbing slides at a density of 106 cells/well. The cells were then fixed with 4% paraformaldehyde for 15 min, permeabilized with 0.3% Triton X-100 for 20 min, and blocked for 1 h. NRF1 antibody (Abcam, Cambridge, UK) as the primary antibody was added on slides and incubated overnight at 4 °C. Rhodamine (TRITC) conjugated goat anti-rabbit IgG (H + L) was then incubated for 1 h at room temperature. Slides were then counterstained for nuclei and sealed with antifade mounting medium with DAPI. Immunofluorescence staining was visualized using a laser scanning confocal microscope (Olympus FV3000, Olympus, Tokyo, Japan) with an excitation wavelength of 561 nm and a 100× silicone oil lens. Relative NRF-1 nuclear/cytosolic fluorescence ratios were quantified using Fiji software v1.4.

### 4.9. Statistical Analysis

Single-cell RNA-sequencing and TCR-sequencing data analysis was performed using R (v4.0.2) for informatics analysis and graphics processing. All experiments were independently repeated at least three times with similar results. Statistical analysis was performed between the two groups using *t*-test (GraphPad Prism 7). To determine the statistical significance of the results, we used the following criteria: * *p* ≤ 0.05, ** *p* ≤ 0.01, *** *p* ≤ 0.001, **** *p* ≤ 0.0001. Differences meeting these criteria were considered statistically significant, while those marked as ns indicated no significant difference.

## Data Availability

The datasets analyzed in this study were from GEO (Gene Expression Omnibus, https://www.ncbi.nlm.nih.gov/geo/, accessed on 4 May 2022) with accession numbers GSE145926 and GSE128033.

## Supplementary Materials

The supporting information can be downloaded at: https://www.mdpi.com/article/10.3390/ijms241612780/s1.
